# Relationship between aerobic capacity and pelvic floor muscles function: a cross-sectional study

**DOI:** 10.1590/1414-431X20175996

**Published:** 2017-09-21

**Authors:** S.P. Jürgensen, A. Borghi-Silva, A.M.F.G. Bastos, G.N. Correia, V.S. Pereira-Baldon, R. Cabiddu, A.M. Catai, P. Driusso

**Affiliations:** 1 Laboratório de Pesquisa em Saúde da Mulher, Departamento de Fisioterapia, Universidade Federal de São Carlos, São Carlos, SP, Brasil; 2 Laboratório de Fisioterapia Cardiopulmonar, Departamento de Fisioterapia, Universidade Federal de São Carlos, São Carlos, SP, Brasil; 3 Laboratório de Fisioterapia Cardiovascular, Departamento de Fisioterapia, Universidade Federal de São Carlos, São Carlos, SP, Brasil

**Keywords:** Functional capacity, Muscle strength, Women, Physical activity, Oxygen consumption

## Abstract

The objective of this study was to evaluate the relationship between aerobic capacity and pelvic floor muscles (PFM) function in adult women. Women aged 18 or over and without urinary dysfunction or other chronic diseases were eligible to participate. They completed the habitual physical activity (HPA) questionnaire, underwent a PFM functional evaluation by palpation and perineometry, and performed a submaximal (between 75 and 85% of maximum heart rate) cardiopulmonary exercise (CPX) test to determine the ventilatory anaerobic threshold (VAT). Forty-one women were included (35±16 years, 75% physically active, 17% very active, and 8% sedentary and 17% presented grade 1 PFM contraction, 31.8% grade 2, 26.8% grade 3, and 24.4% grade 4, according to the modified Oxford Scale). The average PFM contraction pressure obtained by perineometer was 53±26 cmH_2_O and the average oxygen consumption at VAT (VO_2_VAT) obtained from CPX was 14±2 mL·kg^-1^·min^-1^. Significant correlations were found between PFM contraction pressure and VO_2_VAT (r=0.55; P<0.001); between PFM contraction pressure and HPA score (r=0.38; P=0.02); between age and VO_2_VAT (r=-0.25; P=0.049); and between VO_2_VAT and HPA score (r=0.36; P=0.02). An age-adjusted multiple linear regression equation (R^2^=0.32) was derived to estimate VO_2_VAT from the contraction value obtained by perineometer, so that the PFM contraction pressure was able to predict VO_2_VAT. The equation was validated using data from another group of 20 healthy women (33±12 years; PFM contraction: 49±23 cmH_2_O) and no significant difference was found between actual VO_2_VAT and predicted VO_2_VAT (13.1±1.9 *vs* 13.8±2.0 mL·kg^-1^·min^-1^). In conclusion, PFM function is associated with aerobic capacity in healthy women and PFM contraction pressure may be used to estimate VO_2_VAT in this population.

## Introduction

It is well established in the literature that regular physical exercise provides a number of systemic benefits (1), including cardiovascular risk reduction (2) and lung function improvement (3). Physical activity is recommended to preserve health, as well as for the improvement of functional capacity in many chronic diseases, such as hypertension (4), diabetes (5) and metabolic syndrome (6). In the treatment of stress urinary incontinence, pelvic floor muscles (PFM) strength training is considered the gold standard therapeutic approach (7), since urinary incontinence (UI) may be caused by PFM weakness and/or decreased awareness (8). It is now known that women of almost all age groups lack awareness of the pelvic floor muscles, which results in weakness of these muscles, regardless of age (9,10).

It is believed that there may be a relationship between functional capacity and UI, considering that performing exercise programs improves muscle strength, especially of stabilizers and postural muscles, greatly employed during whole body exercise (11). Moreover, the PFM, responsible for the voluntary urinary continence mechanism, are also considered stabilizers and postural muscles, and can be reflexively activated during physical exercise. The PFM are extremely important for the continence mechanism and, in addition, act as a powerful pelvis stabilizer (12). Sapsford and Hodges (13) showed that voluntary abdominal muscles contraction during exercise, especially of the transversus abdominis, results in increased PFM activity in healthy subjects with no history of lower back pain.

Some authors observed that functional decline and the reduction of physical capacity can be associated to the development of UI (14) and that muscular strength reduction is influenced by age (15). In addition, it is known that muscle weakness can increase the risk of falls and that a relationship exists between UI and the risk of falls, due to the urgency to get to the bathroom in time (15,16). However, in a study by Tak et al. (17), institutionalized elderly women participated in a program including weekly sessions over a period of 22 weeks of physical exercises to improve PFM function and simple exercises to improve upper limb mobility, hand function, standing up and sitting down. Results showed no significant reduction in the number of UI cases (17).

Even though the relationship between aerobic capacity and PFM strength has not yet been elucidated, it may be important for the development of new prevention strategies to reduce UI risk through the improvement of physical capacity. We believe it is of fundamental importance to study the relationship between PFM functionality and aerobic capacity, which might be associated with PFM strength, since these muscles also act as postural stabilizers and may therefore influence functional capacity.

In view of this, primary health care strategies, aimed at disease prevention, are being increasingly applied. Therefore, it is important to understand the mechanisms that determine a correlation between the PFM function (an important variable in the prevention of UI) and functional capacity in women who do not present with lower urinary tract dysfunction. Therefore, the objective of the present study was to evaluate the relationship between aerobic capacity and PFM function in adult women without UI or other urinary tract dysfunctions and without conditions affecting the cardiorespiratory system. We hypothesized that the selected population would present a relationship between PFM contraction capacity and oxygen consumption.

## Material and Methods

### Design

The present cross-sectional study was conducted at Federal University of São Carlos (UFSCar), in the Women's Health Research Laboratory (LAMU) and in the Cardiopulmonary Physiotherapy Laboratory (LACAP). Data collection was carried out between December 2012 and January 2014. The sample size calculation was based on a previous study by Müller et al. (18), considering the correlation found between oxygen consumption (VO_2_) and handgrip strength (r=0.74; P<0.001), with a power of 85%, a confidence interval of 95% and a coefficient of determination of 0.15. Based on these parameters, the calculated sample size was 41.

The study received approval by the Centro Universitário Central Paulista (Unicep) Research Ethics Committee (Protocol No. 019/2011). All participants gave written informed consent before data collection began.

### Participants

**Figure 1.**
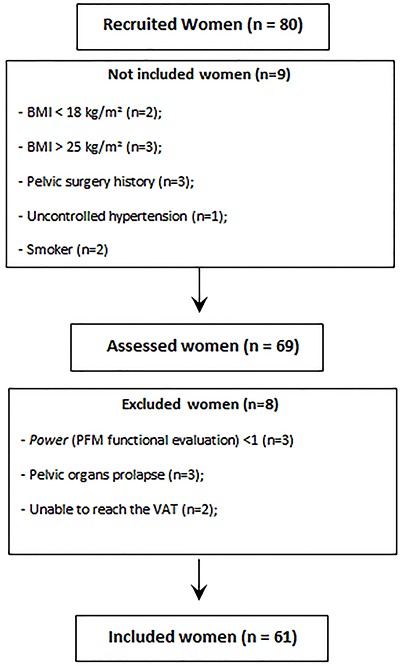
Flowchart of the participants recruited for the present study. BMI: body mass index; PFM: pelvic floor muscles; VAT: ventilatory anaerobic threshold. Twenty of these women were only recruited to evaluate the reliability of the reference equation.

Women were recruited through newspaper advertisements, internet announcements and distribution of flyers to the community. In order to be eligible, women had to be 18 years old or more (9,10), be eutrophic according to body mass index (BMI) (19), and could not present urinary incontinence and/or prolapse of pelvic organs that affect the vaginal opening, according to the Pelvic Organ Prolapse Quantification (POPQ) standard scoring system, nor pelvic surgery history. Women were excluded from the study if they did not present at least a flicker of PFM contraction, i.e. if they presented a grade 0 contraction, according to the 0-5 Modified Oxford Grading Scale (20), and/or if they could not reach the ventilatory anaerobic threshold (VAT) in the cardiopulmonary exercise test (CPX). Women who were currently pregnant, regular smokers, had a history of coronary heart disease, diabetes or cardiac arrhythmia were also excluded from the study (Figure 1).

### Evaluations

All volunteers underwent two evaluations: initial evaluation and CPX, which were carried out with a minimum interval of two days. The day before and on the days of the tests participants were instructed not to consume alcohol and/or stimulants (coffee, tea and others), to avoid heavy meals two hours before the evaluation, to refrain from strenuous exercise, to have an adequate sleep the previous night and to go to the laboratory with comfortable clothes and shoes. In order to avoid the influences of circadian rhythm, all evaluations were performed at the same time of the day (between 12:00 and 6:00 pm).

The initial evaluation consisted of anamnesis, and habitual physical activity (HPA) assessment by Baecke's questionnaire, which has been validated and translated into Brazilian Portuguese (21) and provides a score that classifies the individual as sedentary (below 6) or active (above 6) (22). Participants underwent evaluation of UI symptoms by two structured questions from King's Health Questionnaire (23), and PFM functional assessment by digital palpation and perineometry.

Functional evaluation was performed in the supine position, with hip and knees flexed, according to the protocol proposed by Laycock and Jerwood in 2001 (24). The subjects' strength was classified according to the Modified Oxford Scale, proposed by Laycock (20), which quantifies PFM function as follows: grade 0: no contraction; grade 1: flicker of contraction; grade 2: weak contraction; grade 3: moderate contraction; grade 4: satisfactory contraction; and grade 5: strong contraction.

The PFM contraction pressure was recorded using a Peritron® perineometer (Cardio Design Australia, range from 0 to 300 cmH_2_O); participants remained in the same position they had assumed for the functional evaluation for the introduction of the perineometer vaginal sensor, which had been coated by a sterile non-lubricated male condom (Microtex®), lightly lubricated with an intimate gel (K-Med®). The equipment was then calibrated and volunteers were instructed and verbally motivated to perform three PFM maximal contractions, maintaining each one for 3 s. Participants were instructed to refrain from contraction of abdominal, gluteus and hip adductors muscles (25). The contraction pressure peak value achieved during each contraction was recorded and the average of the three values was calculated.

Finally, with the subjects in the same position, the presence or absence of prolapse was determined by the POP-Q method, which is considered the gold standard by the International Continence Society (26) due to the highly reproducible results.

On the second day of evaluation subjects performed the CPX; a ramp protocol was followed and the test was performed on an electromagnetic braking cycle ergometer (Corival, Lode BV, Netherlands), with the subjects sitting with their knees flexed between 5 and 10 degrees. After 2 min of rest, sitting on the cycle ergometer, a 3-min warm-up period began with a free cycling load (4W). Next, the exercise protocol was initiated with a constant speed of 60 rpm and with power increments determined according to the formula proposed by Wasserman et al. (27), until the submaximal heart rate (HR), previously calculated by Karvonen's formula, was reached (28). The post-test recovery period consisted of 3 min of submaximal cycling, followed by 2 min of rest.

Ventilatory and metabolic variables, as well as the HR, were recorded throughout the whole test, as described below. Volunteers were continuously monitored by electrocardiogram (ECG; Wincardio®, Micromed, Brazil); modified leads MC5, DII, DIII, aVR, aVL and aVF and from V1 to V6 were recorded. Arterial blood pressure (BP) was measured during the pre-test rest period, every 3 min during the test, at the exercise peak and during post-exercise recovery. The tests were conducted by a team of researchers including qualified physiotherapists and exercise physiologist, who constantly monitored the signs and/or symptoms presented by the participants. No adverse events occurred during the test.

Ventilatory and metabolic variables were measured through a portable, computerized ergospirometric device (Oxycon Mobile, Jaeger, Germany). The tidal volume was obtained by a Pitot pneumotachometer (Jager) connected to the system by a face mask, selected according to the size of the participant's face, to be properly adjusted and avoid air leaks. After placing the mask, a few minutes were allowed for the participant's ventilation to become stable. The device provides real-time values for applied power (W), number of cycling rotations per minute (rpm), oxygen consumption (VO_2_), carbon dioxide production (VCO_2_) and pulmonary ventilation (E). The power applied during the exercise protocol was controlled by the system through an interface with the cycle ergometer.

The VAT was derived from VO_2_ and VCO_2_ by implementing the V-slope method (29,30), consisting in plotting VCO_2_ values against VO_2_ values and identifying the change in the curve slope. The method was applied by two researchers, and the average between the values was obtained for each participant.

### Data analysis

Data were tabulated in Excel software and analyzed statistically in Statistica 7.0 software (USA). The Shapiro-Wilk test was applied to verify data normality; the Spearman or Pearson correlation test was applied to quantify the correlation between the variables. Correlations were classified as strong (r>0.70), moderate (r>0.40 and <0.69), weak (r<0.3) or non-existent (r<0.1) (31). Multiple linear regression was used to identify correlations between functional capacity and PFM function parameters; the contraction strength value measured by perineometer presented a moderated correlation with VO_2_VAT and was therefore selected as the predictor to be included in the regression model. The multiple regression model was adjusted for the main confounder (age); the correlation values considered for entry into the regression model were r>0.40. The reliability of the reference equation for the prediction of VO_2_VAT from the contraction strength value was evaluated in another group of 20 healthy women. For each of them, after the evaluations, the contraction strength value and age were included in the equation and the VO_2_VAT was calculated. The predicted value was compared with the value obtained in the CPX, considering an error below 10%. A significance level of 5% (P≤0.05) was adopted.

## Results

Forty-one women participated in the study and their general characteristics are presented in Table 1. Ninety-two percent of the sample was physically active (with 17% presenting a score above 9), while 8% was classified as sedentary, according to the HPA score. According to the Oxford scale (20), 17% of the sample presented only a flicker of contraction, while 83% presented a PFM contraction capacity classified as follows: 31.8%: grade 2, 26.8%: grade 3, 24.4%: grade 4, and none: grade 5.

**Table 1.** Demographic and anthropometric characteristics, and data related to the pelvic floor muscles function and the physical capacity of the studied sample.

Age and anthropometric data	n=41
Age (years)	35±16
Height (cm)	162±6
Body mass (kg)	61±8
BMI (kg/m^2^)	23±3
Physical capacity data	
HPA total score	7.6±1.2
VO_2_VAT CPX (mL·kg^-1^·min^-1^)	13.7±2.2
VE VAT CPX (L/min)	56.0±18.2
PFM functional evaluation data	
Power	2.6±1.0
Perineometer (cmH_2_O)	53.1±25.9

Data are reported as means±SD. N: number of subjects; PFM: pelvic floor muscles; BMI: body mass index; HPA: Habitual Physical Activity questionnaire; VO_2_VAT CPX: oxygen consumption at ventilatory anaerobic threshold of exercise during the cardiopulmonary exercise testing; VE VAT CPX: ventilation at ventilatory anaerobic threshold of exercise during the cardiopulmonary exercise testing; Power: subjective value of digital palpation regarding the graduation of muscle strength.

There was no significant difference between the participants' general characteristics in relation to the degree of PFM contraction (Supplementary Table S1), except for the VO_2_VAT value.

There was a strong correlation between the degree of PFM strength obtained by digital palpation and the value obtained by perineometer (r=0.70; P<0.0001); therefore, the value obtained by digital palpation was not included in the analysis. The three strength values obtained by perineometer during the three contractions were averaged and the mean value was correlated with the following physical capacity variables: HPA score and VO_2_VAT measured in the CPX test (Table 2).

**Table 2.** Results of the correlation analysis between relevant variables.

Variable	r	P value
Digital palpation *vs* perineometer	0.70	<0.001
PFM strength *vs* VO_2_VAT	0.55	<0.05
PFM strength *vs* HPA	0.38	<0.05

Correlation r and P values are presented. PFM: pelvic floor muscles; VO_2_VAT: oxygen consumption at ventilatory anaerobic threshold; HPA: habitual physical activity questionnaire.

**Figure 2.**
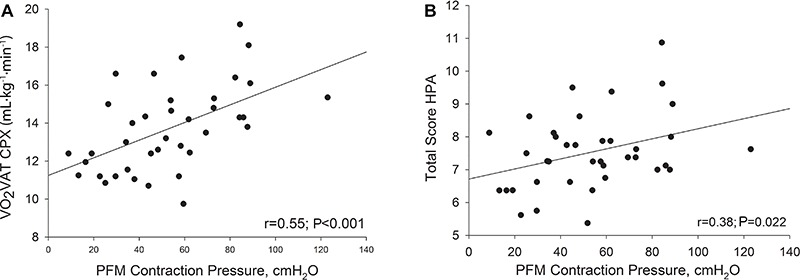
*A*, Correlation between pelvic floor muscles (PFM) contraction pressure and oxygen consumption (VO_2_) at the ventilatory anaerobic threshold (VAT) obtained from the cardiopulmonary exercise test (CPX). *B*, Correlation between PFM contraction pressure and habitual physical activity (HPA) score.

The PFM contraction pressure was significantly and moderately correlated with VO_2_VAT (r=0.55, P<0.05), and significantly and weakly correlated with the HPA score (r=0.38, P<0.05; Figure 2).

Considering these correlations, it was possible to elaborate an age-adjusted multiple linear regression equation to estimate VO_2_VAT from the PFM contraction strength value (Table 3).

**Table 3.** Model for oxygen consumption at ventilatory anaerobic threshold (VO_2_VAT) prediction from pelvic floor muscles (PFM) strength and age.

Variable	Coefficient	Standard error	P value
Constant	12.248	1.068	<0.001
PFM strength (by perineometer, cmH_2_O)	0.0441	0.0123	0.001
Age (years)	-0.0252	0.019	0.16

Reference equation: VO_2_VAT(mL·kg^-1^·min^-1^) = 12.248+(0.0441 × PFM strength) - (0.0252 × Age). Estimated error: 1.916. R^2^=0.32 (R^2^=equation power).

The mean VO_2_VAT value measured in the validation group of 20 women (33±12 years; Perineometer 49±23 cmH_2_O) was not significantly different from the mean VO_2_VAT value predicted by the developed equation (13.1±1.9 *vs* 13.8±2.0), with the actual value representing 95±10% of the predicted value calculated with our reference equation.

## Discussion

The results of the present study showed that the PFM contraction pressure can present significant correlations with physical capacity, assessed by a questionnaire, as well as with the aerobic capacity, assessed by CPX testing. Considering these findings, we propose an age-adjusted equation that allows predicting VO_2_VAT from the PFM contraction value obtained by perineometer.

The relevance of the present study is related to the great physiological importance of the PFM, whose perception and function can be impaired in the female population. A deeper understanding of how the PFM function can be correlated with other factors, such as physical capacity, could represent a starting point to develop better and more efficient prevention and treatment strategies in this population. Furthermore, it is known that UI can arise at different ages, depending on associated factors such as urinary tract infection, obesity, genetic predisposition, menopause, etc. (9,32). By enrolling a group of women with an average age of 35 years, it was possible to carry out a comprehensive study that provided results not influenced by aging. To exclude major influences on the pelvic floor and on the aerobic capacity, only women who presented a BMI within the Brazilian range of normality were included in the present study (19).

All participants underwent symptom-limited submaximal CPX and aerobic capacity was quantified by VO_2_VAT. Oxygen consumption at the anaerobic threshold obtained from the CPX is representative of oxygen consumption during usual activities of daily living. In the present study, we showed that it reflected the PFM strength, with no need for the volunteers to perform a maximal exercise test.

It is known that for other muscles, such as wrist and fingers flexors (33) and quadriceps (34), strength is directly and positively correlated with the oxygen consumption obtained in the CPX. However, the literature mostly reports results obtained from investigations on overall muscle strength (35), which can possibly explain why high correlations were found between muscle strength and physical capacity.

However, to the best of our knowledge, no studies were performed to investigate the association between the PFM contraction strength and the aerobic capacity, assessed by measuring the oxygen consumption. The present study shows that PFM performance could be used for oxygen consumption prediction, as the handgrip strength.

Although considered a small muscle group, the PFM are responsible for stabilizing and sustaining the body, and are therefore directly involved in postural control (11,13). These muscles are in constant activity, and it is known that during activities that require center of mass displacement and coordination (36), their activation may be even higher, which explains the positive correlation between the PFM strength and the VO_2_VAT. Even though the PMF are a small muscle group, the recruitment reflex during stabilization can improve their functionality and that of other muscles.

It is known that sedentary people have reduced aerobic capacity and suffer the effects of physical inactivity, including physical limitations such as reduction in gait speed and a higher risk of falls, primarily due to a decrease in muscle strength and body control (37). When such changes affect the elderly population, the consequences can be worse, since such limitations lead to movement restriction and disability, which can contribute to the onset or worsening of UI.

This can explain why the findings of the present study are in accordance with those presented by Huang et al. (38) who found that among elderly people with UI, those who performed lower levels of physical activity presented severer losses and lower quality of life. However, interestingly, when we divided the participants into groups depending on their PFM muscle strength, we observed that there was no difference in age, i.e., participants with worse scores were not necessarily older. In addition, to the best of our knowledge, no studies including a sample of young women have been conducted so far, which reinforces the importance of the present study. Moreover, our findings highlight the importance of performing regular physical activity also to preserve PFM functionality.

The relationship between PFM function and aerobic capacity reinforces the importance of submaximal exercise programs, which should be combined with specific interventions to preserve these muscles’ integrity and to improve UI in patients with lower urinary tract dysfunction (14,39,40). In addition, it is noteworthy that although the improvement in cardiopulmonary conditioning is associated with better PFM function, there is no evidence that an isolated training can improve symptoms related to UI.

The present study has some limitations that need to be addressed. Although we observed a correlation between aerobic capacity and PFM function, and a standard error of only 1.9 mL·kg^-1^·min^-1^ in the multiple linear regression, age and PFM function explained only 32% of oxygen consumption at VAT. Thus, other unknown factors may directly influence this equation. Moreover, only healthy women were studied in the present investigation. We believe that further studies should be performed including women presenting different BMI values, as well as women with UI or other diseases. Future studies are necessary to evaluate the importance of physical exercise as a way to prevent PFM function loss, which is known to be one of the most important factors that contribute to UI development.

In conclusion, our results showed that PFM functionality was closely related to aerobic capacity in apparently healthy women. Moreover, in the present study, we were able to elaborate an age-adjusted equation that allows predicting VO_2_VAT from PFM strength values obtained by perineometer, which can be used as a tool for the prescription of physical exercise aiming at health promotion.

## Supplementary Material

Click here to view [pdf].
